# Molecular structure‐function relationship of dietary polyphenols for inhibiting VEGF‐induced VEGFR‐2 activity

**DOI:** 10.1002/mnfr.201500407

**Published:** 2015-09-08

**Authors:** Ana B. Cerezo, Mark S. Winterbone, Christina W. A. Moyle, Paul W. Needs, Paul A. Kroon

**Affiliations:** ^1^ Food & Health ProgrammeInstitute of Food ResearchNorwich Research ParkNorwichUK; ^2^ Nutrición y BromatologíaFacultad de FarmaciaUniversidad de SevillaSevillaSpain

**Keywords:** Angiogenesis, Atherosclerosis, Binding affinity, Flavonoids, Phenolics

## Abstract

**Scope:**

We recently reported potent inhibition of VEGF signalling by two flavanols at sub‐micromolar concentrations, mediated by direct binding of the flavanols to VEGF. The aim of this study was to quantify the inhibitory potency and binding affinity of a wide range of dietary polyphenols and determine the structural requirements for VEGF inhibition.

**Methods and results:**

The concentration of polyphenol required to cause 50% inhibition (IC_50_) of VEGF‐dependent VEGFR‐2 activation in HUVECS was determined after pretreating VEGF with polyphenols at various concentations. Binding affinities and binding sites on VEGF were predicted using in‐silico modelling. Ellagic acid and 15 flavonoids had IC_50_ values ≤10 μM while 28 other polyhenols were weak/non‐inhibitors. Structural features associated with potent inhibition included 3‐galloylation, C‐ring C2=C3, total OH, B‐ring catechol, C‐ring 3‐OH of flavonoids. Potency was not associated with polyphenol hydrophobicity. There was a strong correlation between potency of inhibition and binding affinities, and all polyphenols were predicted to bind to a region on VEGF involved in VEGFR‐2 binding.

**Conclusion:**

Specific polyphenols bind directly to a discrete region of VEGF and inhibit VEGF signalling, and this potentially explains the associations between consumption of these polyphenols and CVD risk.

**Abbreviations**
BCA: bicinchoninic acid
CG: catechin gallate
dp 2: apple procyanidin oligomer degree of polymerisation 2
dp 3: apple procyanidin oligomer degree of polymerisation 3
dp 4: apple procyanidin oligomer degree of polymerisation 4
ECG: epicatechin gallate
EGC: epigallocatechin
EGCG: epigallocatechin gallate
EGM‐2: endothelial cell growth medium‐2
HUVECs: human umbilical vein endothelial cells
rhVEGF: recombinant human vascular endothelial growth factor
RIPA buffer: radio‐immunoprecipitation assay buffer
VEGF: vascular endothelial growth factor
VEGFR‐2: vascular endothelial growth factor receptor 2

## 1 Introduction

Plants synthesise a range of polyphenols via the phenylpropanoid biosynthetic pathway and a range of these are consumed as part of human diets in the form of foods and beverages. Flavonoids make up about one third and other polyphenols (mainly simple phenolics) about two thirds of the polyphenols consumed in human diets, with estimates of average daily intakes being around 1200 mg per day 1. There has been considerable interest in the potential health protective properties of dietary polyphenols, supported by epidemiological evidence of an inverse relationship between consumption of flavonoids and other polyphenols and risk of various chronic diseases including coronary heart disease, coronary artery disease, stroke, and certain cancers 2. In addition, there are now hundreds of reports of randomised controlled dietary interventions showing beneficial effects on CVD risk factors and biomarkers of vascular health 3, 4. There are also thousands of scientific reports providing evidence of a multitude of biological activities for polyphenols that have been demonstrated in vitro. However, the majority of the biological activities reported to date remain theoretical because direct evidence of these mechanisms occurring in vivo has not been demonstrated. In the majority of cases, the activities observed in vitro have been in response to supra‐physiological concentrations and/or non‐physiological compounds 5.

To date, the most scientifically robust evidence of health benefits accruing from consumption of dietary polyphenols centres on cardiovascular diseases. The biggest cause of cardiovascular disease is atherosclerosis which is a long‐term process in which artery walls become thickened through the formation of numerous atherosclerotic plaques. This process is dominated by the recruitment of white blood cells to artery walls and the inflammatory responses they produce, and is driven by accumulation of low‐density lipoproteins (which are taken up by the WBCs), differentiation of the white cells into macrophages (pro‐inflammatory) and insufficient removal of fats and cholesterol by high‐density lipoproteins. The growth of atherosclerotic plaques causes thickening of the vascular intima and a crucial consequence of this is to increase the thickness above that capable of supporting diffusion of oxygen and nutrients from the blood. This triggers angiogenesis and the formation of new blood vessels to supply the deeper parts of the plaque with oxygen and nutrients. Angiogenesis, the process resulting in the formation of new blood vessels from pre‐existing ones, plays an important role in the development and destabilisation of atherosclerotic plaques 6, 7, 8. Vascular endothelial growth factor (VEGF) has been shown to be the most important pro‐angiogenic growth factor in humans 9, 10, 11 exerting its angiogenic effects by stimulating VEGF receptor 2 (VEGFR‐2), which is critical for promoting proliferation and differentiation of endothelial cells 11, 12. Indeed, it has been demonstrated directly that VEGF drives the growth of existing plaques in high fat diet‐fed New Zealand White rabbits and hypercholesteraemic ApoE^−/−^ mice 13, 14. Therefore, VEGF‐dependent angiogenesis is an essential process required for growth, healthy functioning of tissues and wound repair (physiological angiogenesis), but can also play a role in pathological processes including atherosclerosis (pathological angiogenesis).

Recently, we reported that two flavan‐3‐ols (epigallocatechin gallate and a procyanidin tetramer), but not others like epicatechin, potently inhibited VEGF‐induced VEGFR‐2 phosphorylation in human umbilical vein endothelial cells (HUVECs), and that potent inhibition only occurred when the VEGF was pre‐incubated with the polyphenols 15. This observation, along with evidence from additional experiments, indicated that the polyphenol‐induced inhibition of VEGF‐mediated activation of VEGFR‐2 was a result of direct binding of the polyphenol(s) to the VEGF protein. This finding was particularly noteworthy because for the first time VEGF was identified as a molecular target that certain polyphenols could interact with directly, and this linked polyphenol biological activities to a receptor (VEGF receptor‐2; VEGFR‐2) 15. In addition, the concentrations of polyphenols required to substantially inhibit VEGFR‐2 signalling were low (in the region of 100–200 nM) and were physiologically relevant.

The overall aim of the research reported here was to assess the ability of a wide range of polyphenols to bind to VEGF and consequently inhibit VEGF‐dependent VEGFR‐2 signalling, and to develop a better understanding of the structural and physicochemical features of polyphenols that contribute to them being potent or impotent inhibitors. We also determined binding affinities for all the polyphenol‐VEGF interactions, and the location of the binding site(s) on the VEGF macromolecule.

## 2 Materials and methods

### 2.1 Cell culture

Human umbilical vein endothelial cells (HUVECs) were obtained from Lonza (Slough, UK), and maintained in Endothelial Cell Growth Medium‐2 (EGM‐2) (Lonza). The cells were cultured at 37ºC in an atmosphere at 5 % CO_2_. All experiments were performed with HUVECs between passages 4 and 5.

### 2.2 Polyphenols for study

The following polyphenols were purchased from Sigma (St. Louis, MO, USA) [CG, ECG, EGC, (+)‐catechin, (−)‐epicatechin, quercetin, morin, 3‐hydroxyflavone, flavone, naringenin, taxifolin, ellagic acid, gallic acid, caffeic acid, chlorogenic acid, resveratrol, genistein, phloridzin, methylgallate, and piceid], Extrasynthese (Lyon, France) [quercetagetin, myricetin, rhamnetin, isorhamnetin, kaempferol, galangin, tamarixetin, luteolin, chrysin, sinensetin, 7‐hydroxyflavone, eriodictyol, isosakuranetin, (+)‐dihydrorobinetin, cyanidin, hyperoside, hirsutrin, quercitrin, rutin], or Apin‐Chemicals (Abingdon, UK) [3′,4′,7,8‐tetrahydroxyflavone].

Isolated single‐mass procyanidin fractions with various degrees of polymerisation (dp 2, dp 3, dp 4) were isolated from apples as previously described 16, 17. EGCG was isolated from green tea as previously described 15. Orobol was synthesised from phloroglucinol and 3,4‐dihydroxyphenylacetic acid using the general procedure of Goto et al. 18 previously used for synthesis of isoflavones.

### 2.3 Polyphenol treatments of VEGF and estimation of IC_50_ values for inhibition of VEGF‐mediated VEGFR‐2 activation

The specific assay used to assess the potency with which polyphenols interact directly with VEGF and inhibit VEGF‐induced VEGFR‐2 phosphorylation has been described elsewhere 15. This assay comprises a pre‐incubation where the polyphenol and the VEGF are mixed and incubated together for 5 min, after which the residual VEGF activity is assessed by adding the mixed polyphenol/VEGF solution to HUVECS for 5 min and the resulting activation of VEGFR‐2 is quantified using an ELISA assay based on a monoclonal antibody specific for the (Phos) Tyr1175 residue in VEGFR‐2.

For each concentration tested, the polyphenol (stocks dissolved in DMSO) was incubated with VEGF (25 ng/mL: human recombinant VEGFA_165_; R&D Systems, Abingdon, UK) in endothelial basal medium (EGM‐2 with no serum or growth factors) for 5 min (final concentration of DMSO ≤ 0.1%). Towards the end of the 5 min incubation period, confluent HUVECs were washed two times with warm PBS in preparation for the addition of the mixture of VEGF and polyphenol. Control incubations included a vehicle control (equivalent concentration of DMSO, ≤ 0.1%) and VEGF (25 ng/mL and final concentration of DMSO ≤ 0.1%) alone. After 5 min exposure of the HUVECs to the pre‐incubated treatments, cells were washed two times with ice‐cold PBS and then lysed with RIPA buffer (radio‐immunoprecipitation assay buffer) containing protease and phosphatase inhibitors. Lysates were transferred to eppendorf tubes and kept on ice for 15 min with periodic vortexing, and subsequently the lysates were clarified by centrifugation at 13 000 *g* for 10 min at 4°C. Supernatants were stored at –80°C until analysis. The total protein content of lysates was determined using a commercially available BCA assay (Sigma, Poole, UK).

Initially, polyphenols were tested at a higher concentration (100 μM) to assess if they had any significant inhibitory activity. Those polyphenols that significantly decreased the phosphorylation of VEGFR‐2 in HUVECs at 100 μM were pre‐incubated with VEGF at a range of concentrations (0.025–200 μM). The specific concentrations selected for each polyphenol were initially estimated from the extent of inhibition at 100 μM. If the inhibition of VEGFR‐2 activation was always less than 50% or always more than 50% then additional assays were conducted such that final datasets for IC_50_ estimations included at least 4 and up to 10 different polyphenol concentrations that spanned above and below the final estimated IC_50_ value.

### 2.4 Phosphorylated VEGFR‐2 ELISA

Quantification of phosphorylated VEGFR‐2 in lysates was determined using a PathScan Phospho‐VEGFR‐2(Tyr1175) sandwich ELISA kit (Cell Signalling Technology, Hitchin, UK), following the manufacturer's instructions. The half inhibitory concentrations (IC_50_) and their confidence intervals were determined by using the log (inhibitor) versus normalised response – variable slope analysis tool in the GraphPad Prism software.

### 2.5 Prediction of polyphenol‐binding sites on VEGF

The crystal structure of VEGF was obtained from the RCBS protein data bank (PDB code: 2vpf, 19). Structures of the ligands (polyphenols) used for docking were obtained from the PubChem chemical library 20. All ligands were subject to binding to VEGF using AutoDock Vina in the PyRX 0.8 Virtual Screening Tool 21. For each ligand the conformer with the lowest free binding energy was taken as the optimal docking conformation.

## 3 Results and discussion

We have previously reported that EGCG from green tea and a tetrameric procyanidin oligomer from apple are potent inhibitors of VEGF‐induced VEGFR‐2 signalling, and achieved this by tightly binding to the VEGF protein and reducing its binding to the VEGFR‐2 receptor 15. The polyphenol‐induced inhibition of VEGF‐induced VEGFR‐2 activation occurred at nanomolar concentrations for these two polyphenols, which may be achieved through diet (IC_50_ values estimated as 88 nM for EGCG and 280 nM for the procyanidin tetramer, Table 1). To further evaluate the potential for polyphenols to inhibit VEGF‐dependent VEGFR‐2 activation through direct interaction with VEGF, we first expanded our investigation into a range of flavanols with different structures and determined what structural features were compatible with potent inhibition. Subsequently, we extended the investigation of structure‐activity relationships to include a range of polyphenols and phenolics with different chemical and structural characteristics. This allowed us to define the key chemical and structural features of polyphenols associated with potent inhibition of VEGF‐dependent VEGFR‐2 activation.

**Table 1:** Chemical structure of polyphenols and their IC_50_ values for inhibiting VEGF in HUVEC cells

Subclass	Name	Additional substitutions			Total	IC_50_ (μM)	Inhibition (%)^a^
		OH	OCH_3_	Others	OH		
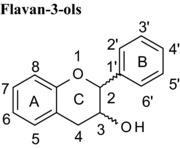							
	EGCG (2R,3R)	5,7,3′,4′,5′		3‐*O*‐Gallate	8	0.088 (0.078–0.100)	
	CG (2R,3S)	5,7,3′,4′		3‐*O*‐Gallate	7	0.094 (0.074–0.097)	
	ECG (2R,3R)	5,7,3′,4′		3‐*O*‐Gallate	7	0.16 (0.150–0.183)	
	EGC (2R,3R)	3,5,7,3′,4′,5′			6	42.9 (36.96–49.97)	
	(+)‐Catechin (2R,3S)	3, 5,7,3′,4′			5	214.7 (178. 2–258.7)	
	(−)‐Epicatechin (2R,3R)	3,5,7,3′,4′			5	N/D	Ineffective (200 μM)
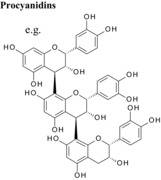							
	Dp 4				20	0.28 (0.266–0.300)	
	Dp 3				15	0.78 (0.679–0.891)	
	Dp 2				10	52.58 (43.85–63.05)	
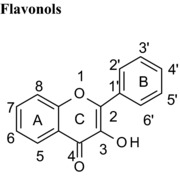							
	Quercetagetin	3,5,6,7,3′,4′			6	0.096 (0.083–0.109)	
	Myricetin	3,5,7,3′,4′,5′			6	0.121(0.102–0.144)	
	Rhamnetin	3,5,3′,4′	7		4	0.547 (0.527–0.568)	
	Quercetin	3,5,7,3′,4′			5	0.754 (0.674–0.842)	
	Morin	3,5,7,2′,4′			5	1.482 (1.357–1.618)	
	Isorhamnetin	3,5,7,4′	3′		4	9.053 (7.676–10.68)	
	Kaempferol	3,5,7,4′			4	9.341 (7.749–11.26)	
	Galangin	3,5,7			3	N/D	85
	Tamarixetin	3,5,7,3′	4′		4	N/D	100 (100 μM)
	3‐hydroxyflavone	3			1	N/D	52.8 (100 μM)
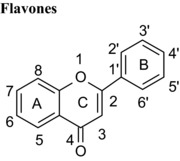							
	Luteolin	5,7,3′,4′			4	7.46 (6.592–8.446)	
	3′,4′,7,8‐tetrahydroxyflavone	7,8,3′,4′			4	N/D	63.2 (100 μM)
	Chrysin	5,7			2	N/D	58.6 (100 μM)
	Sinensetin		5,6,7,3′,4′		5	N/D	Ineffective (100 μM)
	7‐hydroxyflavone	7			1	N/D	Ineffective (100 μM)
	Flavone				0	N/D	Ineffective (100 μM)
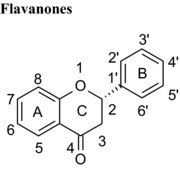							
	Naringenin	5,7,4′			3	N/D	88.5 (100 μM)
	Eriodictyol	5,7,3′,4′			4	N/D	20
	Isosakuranetin	5,7	4′		2	N/D	14.8 (100 μM)
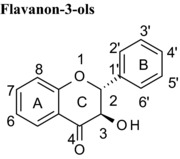							
	(+)‐Dihydrorobinetin	3,7,3′,4′,5′			5	N\D	66
	Taxifolin	3,5,7,3′,4′			5	N/D	12.3 (100 μM)
**Phenolic acids and their derivatives**							
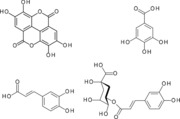							
	Ellagic acid				4	0.230 (0.214–0.247)	
	Gallic acid				4	N/D	32
	Caffeic acid				3	N/D	Ineffective
	Chlorogenic acid				6	N/D	Ineffective
	Methyl gallate				3	N/D	Ineffective (100 μM)
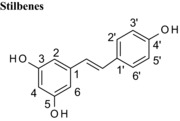							
	Resveratrol	3,5,4′			3	N/D	24
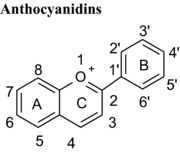							
	Cyanidin	3,5,7,3′,4′			5	1.053 (0.764–1.453)	
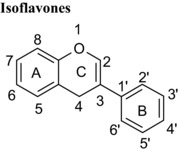							
	Orobol	5,7,3′,4′			4	3.311 (3.047–3.598)	
	Genistein	5,7,4′			3	N/D	22
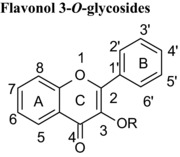							
	Hyperoside	5,7,3′,4′		R = galactoside	8	N/D	Ineffective
	Hirsutrin	5,7,3′,4′		R = glucopyranoside	8	N/D	Ineffective
	Quercitrin	5,7,3′,4′		R = rhamnoside	7	N/D	Ineffective
	Rutin	5,7,3′,4′		R = rutinoside	10	N/D	Ineffective
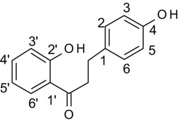							
	Phloridzin	4,2′,4′		6′‐glucoside	7	N/D	Ineffective
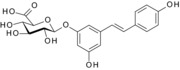							
	Piceid			3‐glucoside	6	N/D	28

a At 50 μM unless indicated otherwise in parentheses.

N/D: non determined.

95% confidence intervals for the IC_50_ values are shown in parentheses.

### 3.1 Concentration‐dependent inhibition of VEGF‐mediated VEGFR‐2 activation and influence of flavan‐3‐ol structure

**Figure 1:**
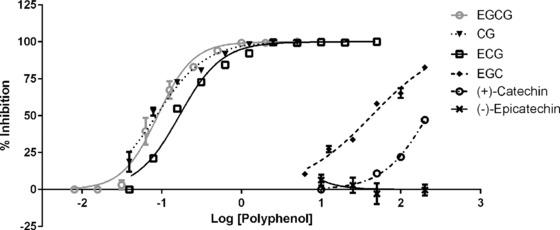
Dose‐response inhibition of VEGFR‐2 activation by pre‐treatment of VEGF with flavan‐3‐ols. VEGF (25 ng/mL) was exposed for 5 min to different polyphenol concentrations (0.008–200 μM) and then applied to HUVECs for 5 min. Phosphorylated VEGFR‐2 was determined by ELISA. Percentage of inhibition represents the difference between VEGFR‐2 phosphorylation of the control (25 ng/mL of VEGF alone) and VEGFR‐2 phosphorylation of the polyphenol treated sample at each concentration. Data are expressed as mean ± SD (*n* = 2 per concentration).

Representative examples of the dose‐dependent inhibition of VEGFR‐2 activation by polyphenol pre‐treated VEGF are shown in Fig. 1. It is immediately obvious from such plots of percent inhibition (y‐axis) plotted against log polyphenol concentration (x‐axis) that some flavanols are very potent inhibitors of VEGF‐mediated VEGFR‐2 activation while others are essentially inactive. The galloylated monomeric catechins epigallocatechin gallate, catechin gallate and epicatechin gallate were all potent inhibitors with estimated IC_50_ values of 0.088, 0.094 and 0.16 μM, respectively. Epigallocatechin and (+)‐catechin were very weak inhibitors (42.9 and 215 μM, respectively) and (−)‐epicatechin was not an inhibitor at even the highest concentration tested (Table 1).

For procyanidins, the tetrameric procyanidin was the most potent, the trimeric dp3 slightly less potent, whereas the procyanidin dimer was a weak inhibitor and the monomers were extremely weak inhibitors. These data show that inhibitory activity increases with increasing degree of polymerisation at least up to dp4, while larger oligomers have not been directly tested. Hydroxylation of the B‐ring also influenced inhibitory activity; e.g. the trihydroxylated B‐ring in EGC conferred stronger inhibitory activity compared to the corresponding dihydroxylated epicatechin.

### 3.2 Galloyl esterification of catechins

3‐Galloyl esters of (+)‐catechin and (−)‐epicatechin were potent inhibitors of VEGF‐mediated VEGFR‐2 activation, whereas the corresponding non‐galloylated compounds were very weak inhibitors (∼2000‐fold higher IC_50_ values), suggesting an important role for the gallic acid ester group in monomeric catechins. It has been reported that gallic acid esters of flavanols such as epigallocatechin (giving rise to EGCG) are potentially very unstable and degrade rapidly in physiological buffers 22. In order to test whether the potent inhibitory nature of (epi)catechin gallic acid esters is a feature of the labile galloyl ester which can potentially release a galloyl group, we tested methylgallate and gallic acid. However, no inhibitory activity could be detected for either gallic acid or methyl gallate. These data suggest that the potent inhibitory activity conferred by the presence of galloyl‐esterification of the 3‐hydroxy group in (epi)catechins contributes to increase the inhibitory potency of the intact molecule rather than facilitate the release of a galloyl group which subsequently interacts with VEGF.

### 3.3 Differences in the C‐ring

Flavonoids are classed according to the chemical structure of the central C‐ring, with the 6 main classes comprising flavan‐3‐ols, flavonols, flavanones, flavones, anthocyanidins and isoflavones, with flavanon‐3‐ols being 3‐hydroxylated derivatives of flavanones. We estimated and compared the IC_50_ values of those compounds with the same hydroxyl group substitution pattern in the A‐ and B‐rings such that the only differences were in the C‐ring. The structural features that contribute to the differences include presence or absence of (1) the C2═C3 double bound (present in quercetin, luteolin, orobol), (2) the 4‐oxo group (present in quercetin, luteolin, eriodictyol and taxifolin) and (3) the positive charge (present in cyanidin), and the B‐ring position in C‐ring. Considering that all these compounds were hydroxylated in the 5‐, 7‐, 3′‐ and 4′‐ positions, the range of inhibitory efficacy was remarkably large, with the most potent being quercetin with an IC_50_ value of 0.75 μM to taxifolin which only inhibited VEGF activity by 12.3% at 100 μM (Table 2).

**Table 2:** Relationships between flavonoid C‐ring structural features and inhibition of VEGF‐induced VEGFR‐2 activation

Compounds	C‐ring structural features					IC_50_ (μM)	Inhibition (%)
	4‐Oxo	C2═C3	+ve charge	B‐ring position	Others		
Quercetin	+	+	–	C2	3‐OH	0.754	
Taxifolin	+	–	–	C2	3‐OH	N/D	12.3 (100 μM)
Catechin	–	–	–	C2	3‐OH	214.7	
Cyanidin	–	–	+	C2	3‐OH	1.05	
Luteolin	+	+	–	C2	–	7.46	
Eriodictyol	+	–	–	C2	–	N/D	20 (50 μM)
Orobol	+	+	–	C3	–	3.311	

N/D: non determined; ‘+’ indicates present, ‘–’ indicates absence.

The presence of the C2═C3 double bond of flavonoids contributes strongly to the inhibition of VEGF‐induced VEGFR‐2 activation, and hydrogenation of the C2═C3 double bond reduces VEGF inhibitory activity significantly. The molecules with saturated C2═C3 bonds (flavanones, flavanols, flavanonols) permit more twisting of the B‐ring in relation to the C‐ring, whereas a C2═C3 double bond increases the p‐conjugation of the bond linking the B‐ and C‐rings, which favors near‐planarity of the two rings. Molecules with near‐planar structures have been shown to more easily enter hydrophobic pockets in proteins 23, 24. Furthermore, quercetin exhibited a >250‐fold lower IC_50_ value compared with (+)‐catechin, whose structure does not contain either the C2═C3 double bound or the 4‐oxo group (Table 2). Therefore, the C2═C3 double bond in conjugation with the 4‐oxo group of flavonoids also makes a significant contribution to the inhibition of VEGF‐induced VEGFR‐2 activation. It appears that planarity of the C‐ring in flavonoids maybe important for binding interaction with proteins.

Although neither cyanidin or (+)‐catechin possess either the C2═C3 double bound or the 4‐oxo group in the C‐ring, the IC_50_ value for cyanidin was 200‐fold lower than (+)‐catechin (Table 2). This is likely to be due to the presence of the positive charge on the C‐ring of cyanidin which is the major difference between the two structures, and if so it suggests that cyanidin may interact with a negatively charged region/residue on the protein which contributes to stabilising the cyanidin‐VEGF complex.

The same trends were observed for other compounds sharing the same OH group substitution patterns but with differences in the C‐ring. For example, luteolin was a more potent inhibitor of VEGF‐induced VEGFR‐2 activation than eriodictyol, which is equivalent in structure except that the C2═C3 double bond is lacking (saturated) (Table 2). Further, myricetin inhibited VEGF activity with an IC_50_>300‐fold lower than EGC, with the main structural difference being the lack of the C2═C3 double bound in conjugation with the 4‐oxo group compared with myricetin (Table 1).

Isoflavones are structural isomers of flavones whose B‐ring is positioned on C3 instead of C2 in the C‐ring. This change ‐ which orientates the B‐ring at an angle approximately 60º around the C‐ring towards the keto group ‐ significantly alters the inhibition of VEGF‐induced VEGFR‐2 activation. For example, the isoflavone orobol exhibited a 2‐fold lower IC_50_ value compared with the flavone luteolin which has an otherwise equivalent structure (Table 1). Therefore, positioning of the B‐ring on C3 of the C‐ring produces more potent VEGF inhibitors than structures where the B‐ring is positioned on C2 of the C‐ring. One plausible explanation is that this serves to position one or more hydroxyl groups in the B‐ring closer to specific amino acid side chains in VEGF and enhances the binding affinity.

### 3.4 Hydroxylation of flavonoids

#### 3.4.1 Hydroxylation on B‐ring of flavonoids

Within the same class, the number of OH groups on the B‐ring of polyphenols affected the VEGF‐inhibitory activity. For example, tri‐hydroxylated B‐ring flavan‐3‐ols such as EGCG (5,7,3′,4′,5′, 3‐gallate) showed IC_50_ values 2‐fold lower than the dihydroxylated B‐ring equivalent ECG (5,7,3′,4′, 3‐gallate), and the IC_50_ for tri‐hydroxylated EGC (3,5,7,3′,4′,5′) was 42.9 μM while di‐hydroxylated epicatechin (3,5,7,3′,4′) was ineffective at 200 μM. Further, the order of potency for the tri‐, di‐, mono‐ and non‐hydroxylated B‐ring series of flavonols were as follows: myricetin (3,5,7,3′,4′,5′) > quercetin (3,5,7,3′,4′) > kaempferol (3,5,7,4′) > galangin (3,5,7). Similarly, the flavone luteolin (5,7,3′,4′) had a reasonably low IC_50_ of 7.46 μM whereas chrysin (5,7) inhibited only 58.6 % of VEGF‐induced VEGFR‐2 activation at 100 μM, and the same effects were observed for isoflavones [compare orobol (5,7,3′,4′; IC50 = 3.31 μM) with genistein (5,7,4′; 20% inhibition at 50 μM)]. VEGF inhibitory activity was affected not just by the number of hydroxyl groups in the B‐ring but also by the position of hydroxylation. For example, although quercetin (3,5,7,3′,4′) and morin (3,5,7,2′,4′) differ only in the position of the two hydroxyl groups on the B‐ring, the IC_50_ of morin was about 2‐fold higher than that of quercetin (Table 1). This observation is consistent with (i) a hydroxyl group in the 3′‐position more strongly interacting (e.g. through H‐bonding) with VEGF than a hydroxyl group in the 2′‐position, or (ii) the chemistry of the catechol group being important for binding to VEGF. Since in all cases increased hydroxylation of the B‐ring conferred increased inhibition of VEGF‐mediated VEGFR‐2 activation, and the position of hydroxylation was also important, hydroxylation of the B‐ring clearly plays an important role in the interaction with the VEGF protein.

#### 3.4.2 Hydroxylation on A‐ring of flavonoids

In contrast to hydroxylation of the B‐ring, the effects of hydroxylation of the A‐ring on VEGF activity depended on the polyphenol class. For flavonols, the IC_50_ value decreased significantly (and potency increased) as the number of OH group substitutions on the A‐ring increased. For example, quercetagetin (3,5,6,7,3′,4′) was a sevenfold more potent VEGF inhibitor than quercetin (3,5,7,3′,4′). There was also a strong effect of position of A‐ring hydroxyl groups. For example, luteolin (5,7,3′,4′) with OH groups in a meta arrangement potently inhibited VEGF activity (IC_50_ = 7.46 μM) while 3′,4′,7,8‐tetrahydroxyflavone with OH groups in an ortho position only inhibited VEGF activity by 63.2 % at 100 μM (Table 1).

#### 3.4.3 Hydroxylation on C‐ring of flavones

Hydroxylation of position 3 of the C‐ring of flavones enhanced the inhibition of VEGF‐induced VEGFR‐2 activation. For example, the IC_50_ value for quercetin (3,5,7,3′,4′) was 10 times lower than that for luteolin (5,7,3′,4′) (Table 1). Similarly, galangin (3,5,7) was a more potent inhibitor than chrysin (5,7) (85% at 50 μM and 58.6% at 100 μM, respectively), and 3‐hydroxyflavone caused 52.8 % inhibition at 100 μM while flavone was ineffective at the same concentration (Table 1). The consistent observation of increased potency of inhibition for 3‐hydroxylated flavonoids strongly suggests that the 3‐hydroxy group is directly involved in binding to VEGF. Research to establish direct evidence of interactions between specific groups on flavonoids with VEGF warrants further investigation.

#### 3.4.4 Total OH groups

**Figure 2:**
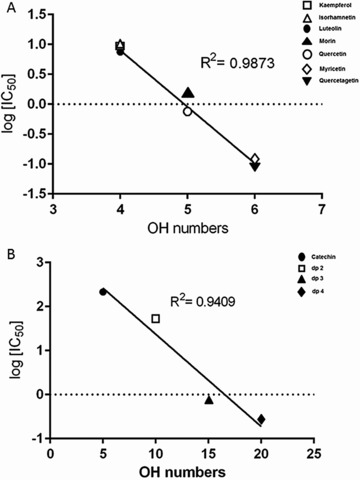
Relationship between the total number of OH groups and the potency of non‐gallated monomeric flavonoids (A) and flavan‐3‐ol monomers and procyanidin oligomers (B) in terms of inhibition of VEGF‐induced VEGFR‐2 activation.

Across the range of compounds studied, there were three major causes of differences in the total number of hydroxyl groups: (i) variations in the number of hydroxyl substitutions on monomeric flavonoids, (ii) 3‐galloyl esterification of flavan‐3‐ols, and (iii) oligomerisation of flavanols. There was a positive correlation (*R* ^2^ = 0.9873) between the total number of hydroxyl substitutions and the potency of a flavonoid in terms of inhibition of VEGF‐induced VEGFR‐2 activation within the non‐gallated monomeric flavonoids (Fig. 2a) except for (+)‐catechin and rhamnetin. The lack of the C2═C3 double bound or the 4‐oxo group in the C‐ring of (+)‐catechin and the methylation on position 7 of rhamnetin have a greater effect on the IC_50_ value than the total number of OH groups ((+)‐catechin: 5 OH groups, IC_50_ = 214.7 μM; rhamnetin: 4 OH groups, IC_50_ = 0.547 μM). As discussed above, 3‐galloylation substantially increases the potency of inhibition of flavan‐3‐ols and this may be related to the additional three hydroxyl groups this adds to the structure. Increasing the degree of polymerisation of flavan‐3‐ols increases the total number of hydroxyl groups and also increases their inhibition potency (Fig. 2b). However, it is not possible to conclude that the increased potency is predominantly due to the increased total number of hydroxyl groups because there is also a substantial change in the total molecular mass as the dp increases.

### 3.5 Conjugation of hydroxyl groups: methylation and glycosylation

Polyphenols are often found with substitution of hydroxyl groups. For example, in plants flavonoids are almost always found O‐linked to sugars through one or more hydroxyl positions on the A, B or C‐rings. Further, during absorption in humans, flavonoid glycosides are deglycosylated by human β‐glucosidases in the gastrointestinal tract 25, 26, 27 and then undergo phase‐2 conjugation in the enterocytes and the liver 28, 29. For some flavonoids such as quercetin (onions, apples, tea), hesperitin (oranges) and (−)‐epicatechin (cocoa, dark chocolate and apples), conjugation is extensive and many phase‐2 conjugates of the flavonoids are found (O‐glucuronides and O‐sulphates of the aglycone and methylated derivatives) 30, 31, 32, 33. In contrast, for other flavonoids and phenolics such as epigallocatechin gallate (green tea) and ellagic acid (pomegranate, raspberries, walnuts), phase‐2 conjugation is hardly detectable and the predominant form of the polyphenol in blood is the aglycone 34. We therefore evaluated whether conjugation and 3‐O‐glycosylations of hydroxyl groups impacted on the ability of the compounds to inhibit VEGF‐induced VEGFR‐2 activation.

Isorhamnetin, which has a methyl group on position 3′, had an estimated 12‐fold higher IC_50_ value than quercetin, which has a hydroxyl group on that position (Table 1). Although IC_50_ values were not calculated for the flavanones, since they were not potent VEGF inhibitors, the same trend was observed for the inhibition percentage of VEGF‐induced VEGFR‐2 activation when naringenin and isosakuranetin were compared at the same concentration; the inhibition percentage of isosakuranetin with a 4′‐methyl substitution was sixfold lower than for naringenin which has a hydroxyl group on that position (Table 1). Thus, methylation of hydroxyl groups on the B‐ring of flavonoids decreases the inhibition of VEGF‐induced VEGFR‐2 activation. In contrast, methylation of hydroxyl groups on the A‐ring did not affect the potential for inhibition of VEGF‐induced VEGFR‐2 activation. Rhamnetin, which has a methyl group on position 7, possessed a similar IC_50_ to quercetin, which presents a hydroxyl group on that position (Table 1). On the other hand, methylation of hydroxyl groups on the A‐ and B‐ring in the same molecule decreased the inhibition of VEGF‐induced VEGFR‐2 activation (sinensetin: methyl groups on position 5,6,7,3′,4′ inhefective at 100 μM; quercetagetin: hydroxyl groups on position 3,5,6,7,3′,4′ IC50 = 0.096 μM).

We also tested whether several 3‐O‐glycosylated forms (galactoside, rutinoside, rhamnoside and glucopyranoside) of quercetin, which was one of the most potent inhibitors (IC_50_: 0.754 μM), modified the inhibition of VEGF‐induced VEGFR‐2 activation. All of the glycosides tested here (hyperoside, hirsutrin, quercitrin and rutin) were completely ineffective at 50 μM. In addition, phloridzin, the 2′‐glucoside form of phloretin was completely ineffective and piceid (resveratrol‐3‐glucoside) inhibited VEGF activity by only 28% at 50 μM (Table 1). These data show that glycosylation has a strong negative impact on the effectiveness of polyphenols as inhibitors of VEGF‐induced VEGFR‐2 activation. There are a number of possible reasons why glycosylation has such a strong effect on VEGF inhibition activity. For example, glycosylation would be expected to introduce a substantial change to the molecular shape and possibly sterically hinder access of key polyphenol substitutions to the VEGF molecule, thus weakening binding to VEGF.

### 3.6 Relationship with polyphenol hydrophobicity

**Figure 3:**
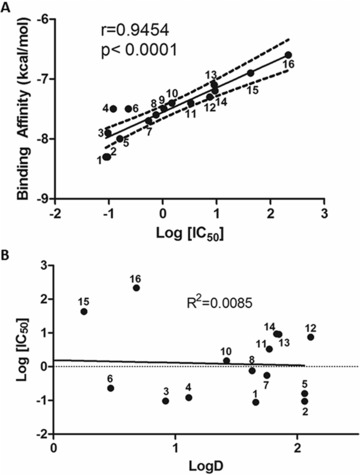
Correlations between (A) the predicted binding constant for binding of monomeric flavonoids to a dimeric VEGF molecule and their corresponding IC_50_ values, and (B) the IC_50_ values and the hydrophobicity of the polyphenols (LogD). The dotted lines indicate the 95% confidence interval. Individual polyphenols are as follows: 1‐EGCG; 2‐CG; 3‐quercetagetin; 4‐myricetin; 5‐ECG; 6‐ellagic acid; 7‐rhamnetin; 8‐quercetin; 9‐cyanidin; 10‐morin; 11‐orobol; 12‐luteolin; 13‐isorhamnetin; 14‐kaempferol; 15‐EGC; 16‐(+)‐catechin. The datapoint for cyanidin in (B) (LogD = –3.130, log IC_50_ = 0.022) is not shown, but it was included in the best‐fit line calculation.

A number of polyphenols are known to interact with proteins in a non‐specific manner, with binding usually driven by hydrophobic or hydrogen bonding interactions between the hydrophobic phenolic rings of the polyphenols and hydrophobic surface regions of proteins 35, 36, 37. However, these non‐specific interactions have only been described at high polyphenol concentrations that are orders of magnitude higher than the IC_50_ values for the VEGF inhibitors reported here (Table 1). Nevertheless, we investigated whether there was a relationship between potency of inhibitory activity and the ‘hydrophobicity’ of the polyphenol, but show that there is no correlation between the polyphenols distribution coefficient (log D_octanol/water_) and the capacity to inhibit VEGF‐dependent VEGFR‐2 activation (IC_50_) (Fig. 3B) (see Supporting Information for LogD data). This observation provides further evidence that the interactions between certain polyphenols and the VEGF protein that cause inhibition of VEGF function are due to site‐specific binding events rather than non‐specific interactions.

### 3.7 Polyphenol‐VEGF binding affinities

**Figure 4:**
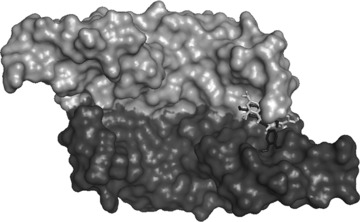
Lowest energy poses of quercetin, luteolin and cyanidin (light grey) and catechin (dark grey) with VEGF attained using AutoDock Vina in the PyRX 0.8 Virtual Screening Tool. Surface representation of VEGF dimer with the individual monomers coloured in light and dark grey.

Binding sites and binding affinities for the lowest energy binding interactions between VEGF and all 44 polyphenols were predicted using in‐silico modelling. The lowest energy poses of polyphenols with dimeric VEGF predicted that all the polyphenols bound to a similar region of VEGF, namely at the end of the groove between the two monomers (Fig. 4 and 12). However, there were some differences in the predicted polyphenol orientation relative to the VEGF protein. For example, the inhibitors quercetin, luteolin and cyanidin were predicted to bind in a different orientation compared to the non‐inhibitor (+)‐catechin (Fig. 4). In vivo, VEGF functions as a dimer, with the two VEGF molecules positioned antiparallel, and this generates two identical ends to the grooves which have been shown to contain the amino acid residues that are involved in VEGF binding to VEGFR‐2 38. We reported previously 15 that EGCG was predicted to bind to VEGF in a groove formed between the two VEGF monomers and next to the VEGFR2 binding site which comprises Tyr36, Ile43 and Ile46 on one side, Asp63 and Glu64 on the other side, with Ser30 and Asp34 forming the bottom of the groove. Here, we report that several other monomeric flavonoids that are inhibitors of VEGF also bind in this groove which is located very close to the binding site for VEGFR2. These monomeric flavonoid inhibitors are predicted to be sufficiently close to the side chains of Phe36, Lys48, Asn62 and Asp63 to be able to interact with them.

The predicted binding affinities for the binding of the various polyphenols to the ends of the groove in VEGF varied widely from –8.3 kcal/mol for catechin gallate and EGCG to –5.2 kcal/mol for methyl gallate (see Supporting Information). Remarkably, there was a very strong correlation (*r* = 0.9445, *p*<0.0001) between the predicted binding affinities and the experimentally determined IC_50_ vales for the set of 15 monomeric polyphenols that exhibited significant inhibitory activity (Fig. 3A). This data provides further evidence to support the notion that the interaction of polyphenols with VEGF is a result of specific rather than non‐specific binding to the protein.

## 4 Concluding remarks

Data presented here show that some specific polyphenols are potent inhibitors of VEGF‐dependent VEGFR‐2 activation, with 11 polyphenols possessing IC_50_ values ≤1 μM, while many other polyphenols are very weak inhibitors or are not inhibitors. The potency of inhibition was strongly related to: the presence of a galloyl group at 3‐positition of flavan‐3‐ols such as EGCG, CG and ECG; the degree of polymerisation of procyanidin oligomers; the presence of a C2═C3 double bound in the C‐ring, especially if conjugated with the 4‐oxo group (flavones and flavonols); the total number of hydroxyl groups on the B‐ring; the presence of the catechol group on the B‐ring; hydroxylation of position 3 on C‐ring; lack of further substitution of hydroxyl groups on the B‐ring. Inhibitory activity was not associated with polyphenol hydrophobicity, and the polyphenols were predicted to bind to a similar region of VEGF which is involved in interactions with its receptor VEGFR‐2, with binding affinities strongly correlated with inhibitory potency. These data are consistent with the notion that potent VEGF inhibitory activity of specific polyphenols is a consequence of specific binding of polyphenols to distinct binding sites on VEGF dimers. It is noteworthy that the polyphenols identified here to have the highest affinities to and to show the strongest inhibition of VEGF activity are those that have most commonly been reported to exhibit anti‐angiogenic and anti‐atherosclerotic activities in a range of in vitro models 39.

*The authors have declared no conflicts of interest*.

## Supporting information

As a service to our authors and readers, this journal provides supporting information supplied by the authors. Such materials are peer reviewed and may be re‐organized for online delivery, but are not copy‐edited or typeset. Technical support issues arising from supporting information (other than missing files) should be addressed to the authors.

Supporting Table
