# Supplementary material for: Molecular structure‐function relationship of dietary polyphenols for inhibiting VEGF‐induced VEGFR‐2 activity
Source: Mol Nutr Food Res. 2015 Sep 8;59(11):2119–31. doi: 10.1002/mnfr.201500407 (PMC4973850; doi:10.1002/mnfr.201500407)
Supplement: Supplementary file 1 — Supporting Table [file MNFR-59-2119-s001.docx]

| Compounds | Binding affinity (kcal/mol) | LogD |
| --- | --- | --- |
| EGCG | -8,3 | 1.66 |
| CG | -8,3 | 2.06 |
| ECG | -8,0 | 2.06 |
| EGC | -6,9 | 0.25 |
| (+)-Catechin | -6.6 | 0.68 |
| (-)-Epicatechin | -6.5 | 0.68 |
| Dp 4 | -9.2 | 1.97 |
| Dp 3 | N/D | 1.51 |
| Dp 2 | N/D | 1.03 |
| Quercetagetin | -7,9 | 0.92 |
| Myricetin | -7,5 | 1.11 |
| Rhamnetin | -7,7 | 1.75 |
| Quercetin | -7,6 | 1.63 |
| Morin | -7,4 | 1.42 |
| Isorhamnetin | -7,1 | 1.85 |
| Kaempferol | -7,2 | 1.83 |
| Galangin | -7.0 | 2.41 |
| Tamarixetin | -7.2 | 1.91 |
| 3-hydroxyflavone | -7.1 | 2.36 |
| Luteolin | -7,3 | 2.11 |
| 3',4',7,8-tetrahydroxyflavone | -7.0 | 1.30 |
| Chrysin | -7.1 | 3.55 |
| Sinensetin | -6.5 | 2.18 |
| 7-hydroxyflavone | -6.8 | 3.38 |
| Flavone | -6.6 | 3.41 |
| Naringenin | -7.2 | 2.34 |
| Eriodictyol | -7.1 | 1.84 |
| Isosakuranetin | -7.2 | 2.86 |
| (+)-Dihydrorobinetin | -7 | 0.41 |
| (+)-Taxifolin | -7.1 | 0.88 |
| Ellagic Acid | -7,5 | 0.47 |
| Gallic acid | -5.6 | -2.42 |
| Caffeic acid | -5.4 | -1.78 |
| Chlorogenic acid | -6.7 | -4.13 |
| Methyl gallate | -5.2 | N/D |
| Resveratrol | -6.7 | 2.59 |
| Cyanidin | -7,5 | -3.13 |
| Orobol | -7,4 | 1.77 |
| Genistein | -6.9 | 2.34 |
| Hyperoside | -7.1 | N/D |
| Hirsutrin | -7 | -0.32 |
| Quercitrin | -7 | 0.14 |
| Rutin | -7.5 | -1.88 |
| Phloridzin | -6.8 | 0.47 |
| Piceid | -7.3 | -4.19 |

**Supporting Information Table 1.** Predicted binding affinity and LogD data for the different polyphenols.

N/D: non determined
